# Artificial Intelligence for Early Detection of Pediatric Eye Diseases Using Mobile Photos

**DOI:** 10.1001/jamanetworkopen.2024.25124

**Published:** 2024-08-06

**Authors:** Qin Shu, Jiali Pang, Zijia Liu, Xiaoyi Liang, Moxin Chen, Zhuoran Tao, Qianwen Liu, Yonglin Guo, Xuefeng Yang, Jinru Ding, Ruiyao Chen, Sujing Wang, Wenjing Li, Guangtao Zhai, Jie Xu, Lin Li

**Affiliations:** 1 Department of Ophthalmology, Shanghai Ninth People’s Hospital, Shanghai Jiao Tong University School of Medicine, Shanghai, China; 2 Shanghai Key Laboratory of Orbital Diseases and Ocular Oncology, Shanghai, China; 3 Shanghai Artificial Intelligence Laboratory, Shanghai, China; 4 Institute of Image Communication and Network Engineering, Shanghai Jiao Tong University, Shanghai, China; 5 Department of Epidemiology and Biostatistics, School of Public Health, Shanghai Jiao Tong University School of Medicine, Shanghai, China

## Abstract

**IMPORTANCE:**

Identifying pediatric eye diseases at an early stage is a worldwide issue. Traditional screening procedures depend on hospitals and ophthalmologists, which are expensive and time-consuming. Using artificial intelligence (AI) to assess children’s eye conditions from mobile photographs could facilitate convenient and early identification of eye disorders in a home setting.

**OBJECTIVE:**

To develop an AI model to identify myopia, strabismus, and ptosis using mobile photographs.

**DESIGN, SETTING, AND PARTICIPANTS:**

This cross-sectional study was conducted at the Department of Ophthalmology of Shanghai Ninth People’s Hospital from October 1, 2022, to September 30, 2023, and included children who were diagnosed with myopia, strabismus, or ptosis.

**MAIN OUTCOMES AND MEASURES:**

A deep learning–based model was developed to identify myopia, strabismus, and ptosis. The performance of the model was assessed using sensitivity, specificity, accuracy, the area under the curve (AUC), positive predictive values (PPV), negative predictive values (NPV), positive likelihood ratios (P-LR), negative likelihood ratios (N-LR), and the F1-score. GradCAM++ was utilized to visually and analytically assess the impact of each region on the model. A sex subgroup analysis and an age subgroup analysis were performed to validate the model’s generalizability.

**RESULTS:**

A total of 1419 images obtained from 476 patients (225 female [47.27%]; 299 [62.82%] aged between 6 and 12 years) were used to build the model. Among them, 946 monocular images were used to identify myopia and ptosis, and 473 binocular images were used to identify strabismus. The model demonstrated good sensitivity in detecting myopia (0.84 [95% CI, 0.82-0.87]), strabismus (0.73 [95% CI, 0.70-0.77]), and ptosis (0.85 [95% CI, 0.82-0.87]). The model showed comparable performance in identifying eye disorders in both female and male children during sex subgroup analysis. There were differences in identifying eye disorders among different age subgroups.

**CONCLUSIONS AND RELEVANCE:**

In this cross-sectional study, the AI model demonstrated strong performance in accurately identifying myopia, strabismus, and ptosis using only smartphone images. These results suggest that such a model could facilitate the early detection of pediatric eye diseases in a convenient manner at home.

## Key Points

**Question:**

Can artificial intelligence (AI) models help to identify pediatric eye diseases such as myopia, strabismus, and ptosis early by analyzing mobile photographs?

**Findings:**

In this cross-sectional study, a deep learning–based model was developed for early identification of myopia, strabismus, and ptosis using 1419 facial photographs from 476 individuals. The AI model demonstrated high accuracy in detecting all of these pediatric eye diseases.

**Meaning:**

These results suggest that AI has substantial potential for the early detection of pediatric eye diseases using mobile photographs taken at home.

## Teaser

This cross-sectional study assesses whether a newly developed artificial intelligence (AI) model can accurately identify myopia, strabismus, and ptosis in children using mobile photographs.

## Introduction

Children’s health is a worldwide concern, and eye health in children has received a lot of attention. Approximately 50% of schoolchildren under the age of 18 years in China are affected by myopia.^1,2^ Strabismus occurs in 1.19% to 5.65% of children^3,4,5,6,7^ and ptosis affects around 0.18% to 1.41% of children.^8,9,10^

Myopia, strabismus, and ptosis are common eye problems in children that can greatly damage their visual health, overall well-being, and development.^11,12,13,14^ Once myopia occurs it is irreversible, and early myopia has the potential to develop into high myopia if it is not recognized and controlled. While high myopia is often associated with several severe eye issues, including retinal detachment, myopic macular degeneration, choroidal neovascularization, glaucoma, and cataracts, which can lead to substantial visual impairment and potential blindness.^15,16,17,18,19^ Strabismus and ptosis do not just impair a patient’s appearance, they may also affect the development of the visual system, especially during childhood, which is a critical period of visual development.^5,8^

Early screening and identification of these diseases are essential for successful management and therapy. Screening for various eye illnesses is primarily conducted in hospitals by expert ophthalmologists, causing delays in screening, diagnosis, and treatment.^20,21,22,23,24,25,26,27^ A useful screening approach is needed to allow parents to do early screening at home and overcome these limitations.

The emergence of artificial intelligence (AI) has transformed the identification of eye diseases by overcoming limitations related to time and spatial distance.^28,29,30^ Deep learning has effectively diagnosed several eye disorders such as myopia,^31^ strabismus,^32,33^ and ptosis.^34^ Current AI techniques for myopia diagnosis use optical coherence tomography macular images, which are not commonly used for identifying myopia.^35,36^ Detections for strabismus and ptosis are made using ocular appearance photographs along with additional labeling or measurements.^34,37^ Identifying strabismus and ptosis merely based on ocular appearance photographs is exceedingly difficult.

This study aims to create an AI model that uses mobile photographs to predict myopia, strabismus, and ptosis in children and adolescents. A multifunctional model would offer parents a user-friendly tool to identify numerous scenarios simultaneously, enhancing accuracy and convenience.

## Method

### Participants and Data Source

This cross-sectional study was conducted from October 1, 2022, to September 30, 2023, to enroll participants at the Ophthalmology Department of Shanghai Ninth People’s Hospital, School of Medicine, Shanghai Jiao Tong University. The study followed a predetermined protocol that was approved by the Ethics Committee of Shanghai Ninth People’s Hospital, School of Medicine, Shanghai Jiao Tong University, and adhered to the tenets of the Declaration of Helsinki.

The study included participants who were less than or equal to 18 years of age, had been diagnosed with myopia, strabismus, or ptosis after an ophthalmologic examination at our hospital, had complete medical records and examination data, were able to cooperate during facial image acquisition to obtain clear images, and had provided informed consent from both themselves and their guardians. Exclusion criteria included those with ocular conditions affecting image capture, severe facial abnormalities, history of strabismus or ptosis reconstructive surgery, and psychological or psychiatric issues.

Participants’ faces were photographed in the clinic room under lighting conditions of 300 to 500 lux. The images were taken using a smartphone from a distance of 1.64 inches from the patient. The patient was instructed to remove their spectacles, maintain their head upright, and stare straight ahead.

### Data Labeling

All participants received an ophthalmologic examination and were diagnosed by a professional ophthalmologist. Myopia diagnosis primarily relies on cycloplegic computerized optometry examination. If the spherical equivalent refraction is less than or equal to −0.50D (the algebraic total in diopters, sphere plus ½ cylinder), we classify the participant as myopic. The diagnosis of strabismus relied mostly on the prism and alternate cover test. Typically, when the frontalis muscle is not active, individuals open their eyes and gaze straight ahead, with the top eyelid margin covering the upper edge of the cornea by a maximum of 2 mm. A diagnosis of ptosis was made if the measurement exceeded 2 mm.

During the data labeling phase, we integrated the individuals’ ophthalmologic diagnosis data with basic information such as name, sex, and age to classify facial photographs. Participants with myopia and ptosis were labeled monocularly, while patients with strabismus were labeled binocularly. We captured screenshots of each participant’s right eye, left eye, and both eyes to align with their medical history data for analysis.

### AI Model Construction

The study used the ConvNeXt^38^ deep learning network to independently detect myopia, strabismus, and ptosis. Before inputting the images into the network, the original images were cropped. Specifically, for myopia and ptosis data, we obtained single-eye local images using a square bounding box. For strabismus, we obtained dual-eye local images using a rectangular bounding box. Subsequently, we resized the single-eye images to 256 × 256 pixels and the dual-eye images to 128 × 512 pixels.

The network architecture consists of 5 main components, as illustrated in eFigure 1 in Supplement 1. The first component includes a convolutional layer, a layer normalization operation, and 3 convolutional neural network (CNN) blocks. The next 3 components each start with down sampling, followed by 3, 9, and 3 CNN blocks, respectively.^39^ The final component, a fully connected layer, outputs the probabilities of the target categories.

Furthermore, we used pretraining weights to fine-tune our network as a result of the limited quantity of data available. The pretraining weights are acquired through prior training on the ImageNet-1k dataset.^40^ We initialized the model with all the pretraining weights, except for the classification layer. Additionally, a data augmentation approach is used to reduce overfitting during training. The training data was subjected to random horizontal and vertical flipping before being fed into the network.

Moreover, the cross-entropy loss function was used to quantify the loss between each iteration’s outcome and the corresponding label during the iteration process. The network parameters were optimized using an Adam optimizer^41^ with a learning rate of 0.0001. The batch size was set at 16. The training was conducted throughout 200 epochs. The network was constructed using the Python package Pytorch (version 2.0.1)^42^ with Python version 3.10.13. The arithmetic required for model development was performed using an NVIDIA RTX 4090 graphics card.

### AI Model Evaluation

We evaluated the AI model’s performance in identifying myopia, strabismus, and ptosis with assessment metrics such as sensitivity, specificity, accuracy, the area under the curve (AUC), positive predictive values (PPV), negative predictive values (NPV), positive likelihood ratio (P-LR), negative likelihood ratio (N-LR), and F1-score based on 5-fold cross-validations. We assessed the model’s generalizability in sex and age subgroups by calculating the metrics. The age categories were segmented into 3 groups: 0 to 5 years, 6 to 12 years, and 13 to 18 years. Gradient-weighted class activation maps were used to generate heatmaps for the photos that were identified as positive by the AI model.^43^

### Statistical Analysis

A demographic table was generated to describe patient distribution. We summarized the number and percentage of patients with each disease regarding their sex, age group, and number of diseases. Besides, body mass index (BMI) was summarized using the median and IQR as it did not follow a normal distribution (D’Agostino and Pearson test *P* value <.001). The test was conducted by the Scipy package in Python. Mean values and 95% CIs of each evaluation metric were calculated for assessment. For all statistical tests, 2-sided *P* < .05 was considered statistically significant. Data analysis was performed using Python version 3.10.13 (Python Software Foundation). All metrics were calculated through Python sklearn, Numpy, and Pandas packages.

## Results

### Data Characteristics

**Figure 1.**
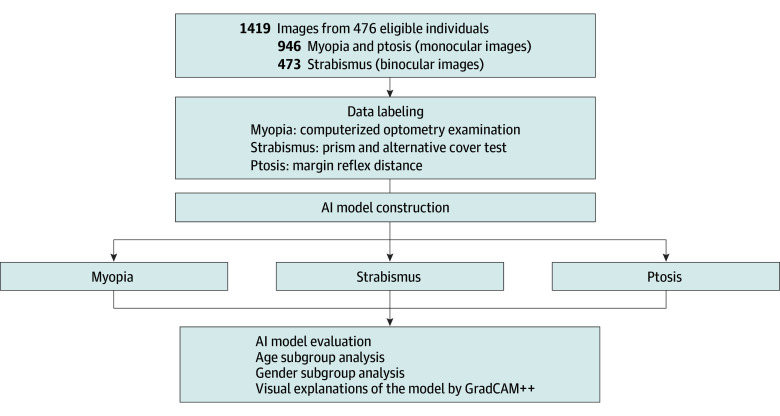
Overview of Study Design AI indicates artificial intelligence.

Among 476 patients who fulfilled the study criteria, 251 (52.73%) were male and 299 [62.82%] were between 6 and 12 years of age (Table 1). We collected 1419 images from these patients for model construction: 946 monocular images for estimating myopia and ptosis, and 473 binocular images for identifying strabismus (Figure 1).

**Table 1.** Clinical Characteristics of the Dataset

Clinical values	Patients, No. (%)			
	Total (N = 476)	Myopia (n = 251)	Strabismus (n = 180)	Ptosis (n = 171)
Sex				
Male	251 (52.73)	131 (52.19)	86 (47.78)	97 (56.73)
Female	225 (47.27)	120 (47.81)	94 (52.22)	74 (43.27)
Age group, y				
0-5	94 (19.75)	10 (3.98)	36 (20.00)	61 (35.67)
6-12	299 (62.82)	177 (70.52)	111 (61.67)	89 (52.05)
13-18	82 (17.23)	64 (25.50)	33 (18.33)	21 (12.28)
BMI, median (IQR)	16.81 (15.31-19.39)	17.42 (15.38-20.08)	16.57 (15.33-19.64)	16.75 (15.12-19.64)
No. of eye diseases				
1	331 (69.54)	137 (54.58)	74 (41.11)	120 (70.18)
2	125 (26.26)	107 (42.63)	99 (55.00)	44 (25.73)
3	7 (1.47)	7 (2.79)	7 (3.89)	7 (4.09)

Abbreviation: BMI, body mass index (calculated as weight in kilograms divided by height in meters squared).

There were 251 patients with myopia, 180 patients with strabismus, and 171 patients with ptosis. According to Table 1, male and female participants were distributed similarly in these 3 diseases. Besides, some patients had 2 or all of the diseases; 7 patients (1.47%) had myopia, strabismus, and ptosis; 107 patients (42.63%) had myopia and another disease, 99 patients (55.00%) had strabismus and another disease, and 44 patients (25.73%) had ptosis and another disease (Table 1).

### Model Performance

We assessed the performance of the AI model for each pediatric eye disease using metrics such as sensitivity, specificity, accuracy, AUC, PPV, NPV, P-LR, N-LR, and F1-score (Table 2). The model demonstrated good performance in detecting children’s eye diseases from mobile photographs, with accuracies for identifying the 3 eye diseases exceeding 0.80. The model excelled in detecting ptosis, demonstrating a sensitivity of 0.85 (95% CI, 0.82-0.87), specificity of 0.95 (95% CI, 0.93-0.97), accuracy of 0.92 (95% CI, 0.91-0.93), AUC of 0.94 (95% CI, 0.93-0.96), and F1-score of 0.87 (95% CI, 0.85-0.88). The model demonstrated strong performance in identifying myopia, with a sensitivity of 0.84 (95% CI, 0.82-0.87), specificity of 0.76 (95% CI, 0.73-0.80), accuracy of 0.80 (95% CI, 0.78-0.81), AUC of 0.84 (95% CI, 0.83-0.85), and F1-score of 0.77 (95% CI, 0.76-0.78). As for strabismus, the model performed well with a sensitivity of 0.73 (95% CI, 0.70-0.77), specificity of 0.85 (95% CI, 0.84-0.86), accuracy of 0.80 (95% CI, 0.79-0.82), AUC of 0.83 (95% CI, 0.82-0.85), and F1-score of 0.74 (95% CI, 0.72-0.76). Receiver operating characteristic (ROC) curves were shown in eFigure 2 in Supplement 1. Confusion matrices showed the classification details of myopia, strabismus, and ptosis in testing datasets (eFigure 3 in Supplement 1).

**Table 2.** Model Performance in Predicting Myopia, Strabismus, and Ptosis

	Sensitivity (95% CI)	Specificity (95% CI)	Accuracy (95% CI)	AUC (95% CI)	PPV (95% CI)	NPV (95% CI)	P-LR (95% CI)	N-LR (95% CI)	F1-score (95% CI)
Myopia	0.84 (0.82-0.87)	0.76 (0.73-0.80)	0.80 (0.78-0.81)	0.84 (0.83-0.85)	0.71 (0.69-0.74)	0.88 (0.86-0.89)	3.62 (3.19-4.06)	0.20 (0.18-0.23)	0.77 (0.76-0.78)
Strabismus	0.73 (0.70-0.77)	0.85 (0.84-0.86)	0.80 (0.79-0.82)	0.83 (0.82-0.85)	0.75 (0.74-0.76)	0.84 (0.82-0.86)	4.86 (4.56-5.15)	0.32 (0.27-0.36)	0.74 (0.72-0.76)
Ptosis	0.85 (0.82-0.87)	0.95 (0.93-0.97)	0.92 (0.91-0.93)	0.94 (0.93-0.96)	0.89 (0.84-0.93)	0.94 (0.93-0.95)	23.79 (10.56-37.02)	0.16 (0.14-0.18)	0.87 (0.85-0.88)

Abbreviations: AUC, area under the curve; N-LR, negative likelihood ratio; NPV, negative predictive values; P-LR, positive likelihood ratio; PPV, positive predictive values.

**Figure 2.**
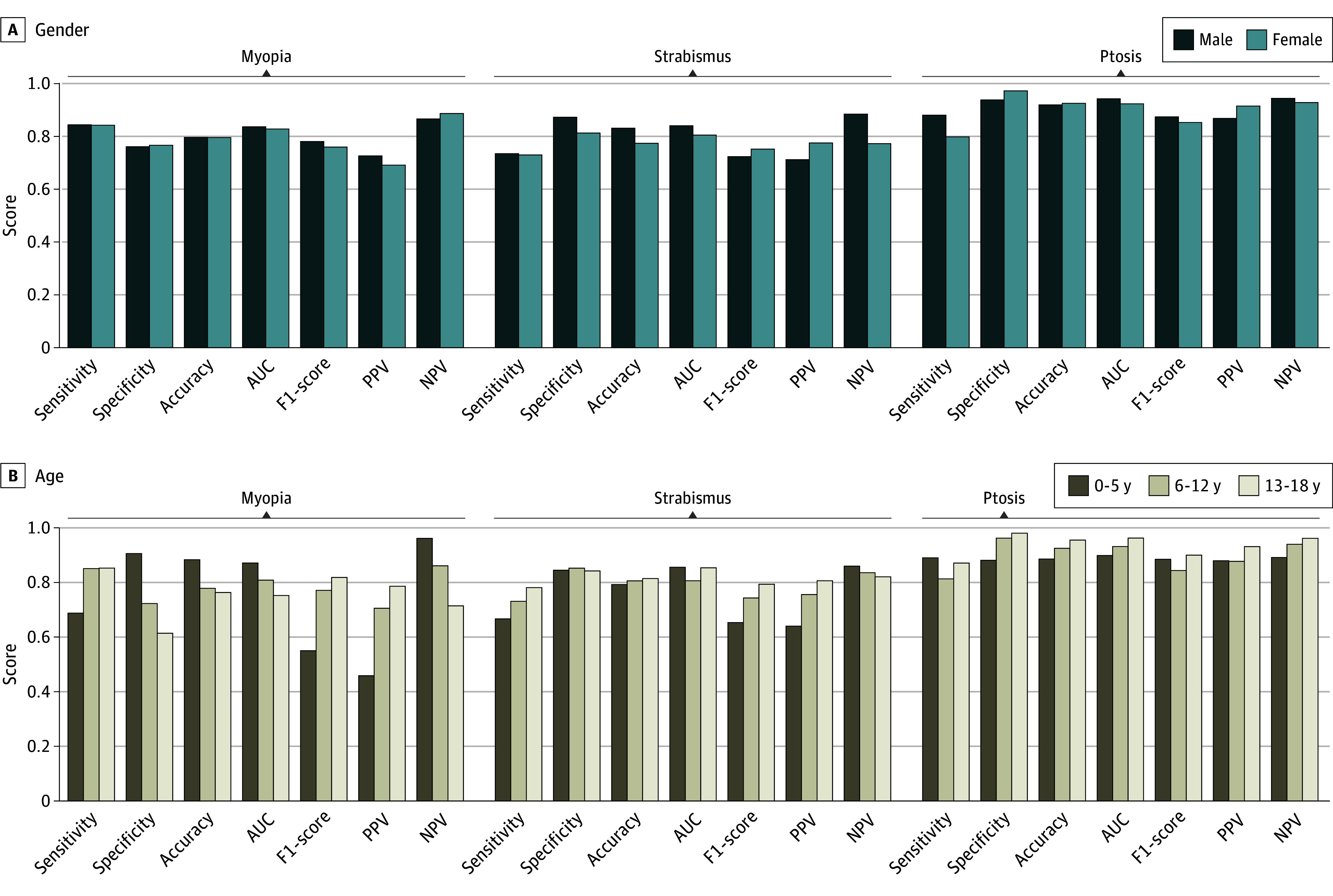
Model Performance Evaluated by Subgroup Analysis A, Barplot of the evaluation metrics based on sex groups: male and female. B, Barplot of the evaluation metrics based on age groups: 0 to 5 years, 6 to 12 years, and 13 to 18 years. AUC indicates area under the curve; NPV, negative predictive value; PPV, positive predictive value.

We analyzed the generalizability of our AI model for detecting pediatric eye diseases by calculating evaluation metrics in sex and age subgroups. The model showed comparable performance in identifying myopia, strabismus, and ptosis for both females and males within different sex groupings (Figure 2A). When analyzing the model’s performance, differences in sensitivity and specificity were observed among different age subgroups. The model had good sensitivity for identifying myopia in the age subgroups of 13 to 18 years and 6 to 12 years (both approximately 0.85), whereas the sensitivity was relatively low at 0.69 for the age subgroup of 0 to 5 years (Figure 2B). The model had the maximum sensitivity of 0.78 in identifying strabismus for the age subgroup of 13 to 18 years and the lowest sensitivity of 0.67 for the subgroup of 0 to 5 years (Figure 2B). When evaluating different age groups, the model shows the most consistent performance in detecting ptosis. Overall, the model achieved relatively high sensitivity in detecting these 3 eye diseases in different age groups and sex groups.

### Model Interpretation by Heatmaps

**Figure 3.**
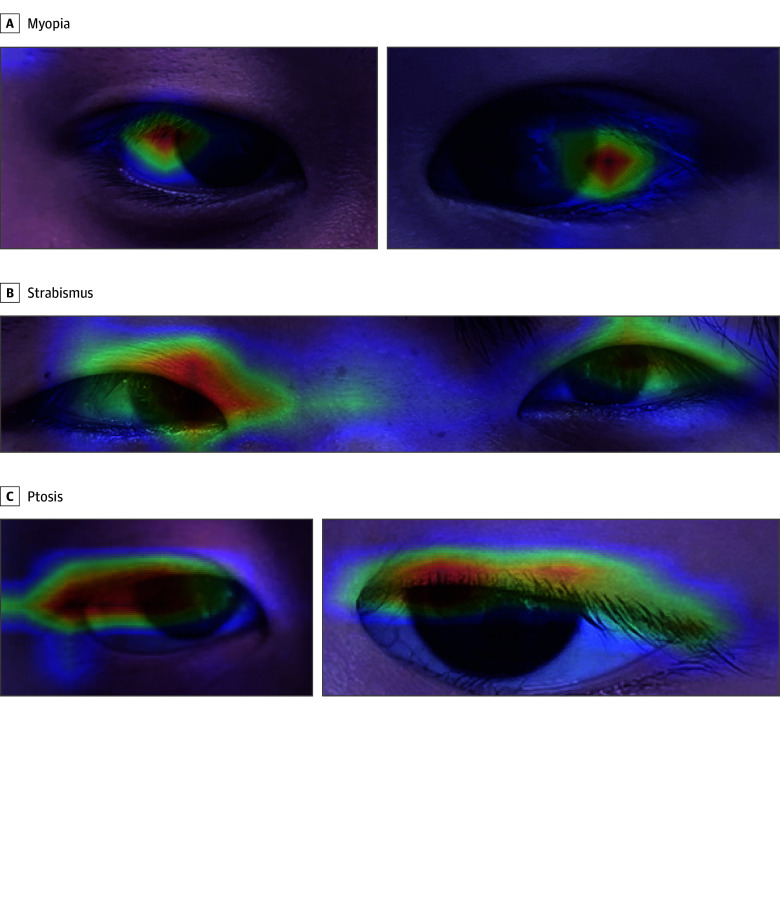
Heatmaps for Myopia, Strabismus, and Ptosis A, Two examples of myopia (left eye and right eye) detected by the artificial intelligence (AI) model. B, An example of strabismus detected by the AI model. C, Two examples of ptosis (left eye and right eye) detected by the AI model.

We used GradCAM++ to evaluate the weight of different regions in the eye images on the classification outcomes of the AI model identifying pediatric eye disorders. The importance of individual pixels in picture categorization is demonstrated through a heatmap created by combining the feature maps based on the network weights. Regions of greater significance are represented by the use of warmer colors in the heatmap. The weight of eye regions on the model’s predictions of myopia, strabismus, and ptosis varies, as illustrated in Figure 3. The most influential regions in identifying myopia are the sclera located at the temporal edge of the pupil in the affected eyes. The most important locations for identifying strabismus are located on the side of the affected eyes. The most important areas for identifying ptosis are located on the eyelids.

## Discussion

In this study, 476 patients’ faces from a total of 1419 photographs were analyzed to develop models that could detect myopia, strabismus, and ptosis all at once; 946 monocular photographs were used to construct a detection model for myopia and ptosis, and 473 binocular photographs were used to build a detection model for strabismus. The AI model demonstrated high accuracy in detecting myopia, strabismus, and ptosis, with accuracies of 0.80 (95% CI, 0.78-0.81), 0.80 (95% CI, 0.79-0.82), and 0.92 (95% CI, 0.91-0.93), respectively. The model’s performance for these 3 diseases was constant across sex subgroups; however, changes were noted in age subgroups, showing increasing sensitivity for myopia and strabismus with age. These results suggest that AI prediction models utilizing smartphone photographs may identify eye diseases in children and adolescents, providing a handy and early diagnostic tool for families to use at home (eFigure 4 in Supplement 1).

### Myopia

Myopia, the most common eye disease among children and teenagers, has increased across numerous countries and areas worldwide, emerging as a global public health concern.^44,45,46^ Children who develop myopia at a younger age (6-8 years) will have a longer period of rapid eye growth, allowing their myopia to progress more than those who get myopia later, at 12 years of age or older, when growth is slower. Younger individuals are more susceptible to developing high myopia (≤−6.0 D of correction) in the future. Delaying the start of myopia or decreasing the speed at which myopia advances can lower the chances of children developing high myopia in the future, and early identification of myopia is important for this.

Currently, Myopia screening tools are primarily found in hospitals, with no available options for home screening. This study has developed a detection model that can be integrated into a mobile phone for parents to do initial myopia screening on children and adolescents at home. The study demonstrated a mean 0.80 accuracy in identifying myopia, indicating that 8 out of 10 individuals could be correctly identified as myopic. This echoes the outcomes of a prior study that used a singular myopia detection model based on facial photographs.^31^ The model demonstrated greater prediction sensitivity in older age groups and was more precise in identifying individuals with myopia. This could be related to the myopia progression with age, and the model can more easily identify it as the myopia level rises.

### Strabismus

Strabismus adversely affects the patient’s appearance and visual function, substantially diminishing vision and quality of life, and perhaps resulting in psychological distress and illness.^5,47,48,49,50^ The present screening methods are laborious, time-consuming, and necessitate expert clinicians. This study uses face recognition and deep learning techniques to construct a detection model for strabismus screening, aiming to decrease the demands on clinicians, instruments, and time, enhance result reliability, and enable home self-test screening. The strabismus detection model has an overall accuracy of 0.80 (95% CI, 0.79-0.82) and a sensitivity of 0.73 (95% CI, 0.70-0.77), demonstrating its great capability to identify children and adolescents with strabismus accurately. However, compared with the strabismus detection model built in previous studies, the accuracy and sensitivity are slightly lower.^32,51^ This could be attributed to the inclusion of just 1 photograph of a single participant in this study. To enhance the precision of the strabismus detection model, we might gather images of various eye locations and apply random changes to the images for data augmentation in upcoming analyses. During the age subgroup analysis, we observed that the accuracy of model recognition was stable across age groups.

### Ptosis

Children with ptosis commonly exhibit a drooping eyelid that covers the pupil in one or both eyes. This condition typically manifests in early childhood and preschool age.^52^ During this crucial period, children’s visual development is at risk, and extended vision obstruction can result in amblyopia, inadequate eye growth, and various other issues.^8^ Ptosis is the most noticeable of the 3 disorders in this study, yet it may be challenging for parents without medical knowledge to identify it correctly. Creating a diagnostic tool for at-home ptosis screening is crucial. Research has been carried out in this field, and the sensitivity of the screening models created varied from 0.74 to 0.92.^53,54,55^ The ptosis screening model created in this work had an accuracy of 0.92 (95% CI, 0.91-0.93) and a sensitivity of 0.85 (95% CI, 0.82-0.87), suggesting excellent screening capabilities. Due to its consistent performance, there was no notable variation in the detection accuracy of subgroup analysis based on sex and age.

### Strengths and Limitations

To our knowledge, this study is the first to use face photographs of children and adolescents to predict myopia, strabismus, and ptosis simultaneously. Home screening for 3 common eye diseases in children and adolescents may be performed with a smartphone application (eFigure 4 in Supplement 1), which helps reduce the burden on health care. Nevertheless, there are limitations of this study. First, this is a single-center cross-sectional study with a small sample size, suggesting the need for a multicenter investigation to enhance the algorithm’s generalizability. When taking photographs, only 1 snapshot is gathered for each individual in this investigation, which restricts the algorithm’s capabilities due to insufficient information. Collecting patients’ images from various perspectives can enhance the algorithm’s performance. In addition, the sample sizes of the 3 different diseases in this study were different, with myopia having the largest sample size and strabismus having the smallest sample size, which may be one of the potential reasons for the model’s low sensitivity in strabismus detection. Increasing the number of facial photographs of strabismus patients could help the model improve its performance in strabismus detection. The existing model is a binary classification model, although the severity of myopia, strabismus, and ptosis differs among patients. In the future, we aim to develop a model capable of assessing the severity of myopia, strabismus, and ptosis.

## Conclusions

This cross-sectional study found that the detection model using AI showed strong performance in accurately identifying myopia, strabismus, and ptosis using only smartphone images. These results suggest that it can assist families in screening children for myopia, strabismus, and ptosis, facilitating early identification and reducing the risk of visual function loss and severe problems due to delayed screening. Moreover, using such information can help achieve a more equitable allocation of limited medical resources. This is critical to the advancement of global health standards.

## Notes

Supplement 1. **eFigure 1.** Framework of the AI Model **eFigure 2.** Performance of Myopia, Strabismus, and Ptosis Detection Models **eFigure 3.** Comparison of the Classification Performance of the Model on the Testing Dataset **eFigure 4.** Pediatric Eye Diseases Detection APP Design

Supplement 2.
Data Sharing Statement
