# Supplementary material for: Artificial Intelligence for Early Detection of Pediatric Eye Diseases Using Mobile Photos
Source: JAMA Netw Open. 2024 Aug 6;7(8):e2425124. doi: 10.1001/jamanetworkopen.2024.25124 (PMC11304122; doi:10.1001/jamanetworkopen.2024.25124)
Supplement: Supplement 1. — eFigure 1. Framework of the AI Model eFigure 2. Performance of Myopia, Strabismus, and Ptosis Detection Models eFigure 3. Comparison of the Classification Performance of the Model on the Testing Dataset eFigure 4. Pediatric Eye Diseases Detection APP Design [file jamanetwopen-e2425124-s001.pdf]

Supplemental Online Content

Shu Q, Pang J, Liu Z, et al. Artificial intelligence for early detection of pediatric eye diseases using mobile photos. *JAMA Netw Open.* 2024;7(8):e2425124. doi:10.1001/jamanetworkopen.2024.25124

eFigure 1. Framework of the AI Model

eFigure 2. Performance of Myopia, Strabismus, and Ptosis Detection Models

eFigure 3. Comparison of the Classification Performance of the Model on the Testing Dataset

eFigure 4. Pediatric Eye Diseases Detection APP Design

This supplemental material has been provided by the authors to give readers additional information about their work.

© 2024 Shu Q et al. *JAMA Network Open.*

eFigure 1. Framework of the AI model.

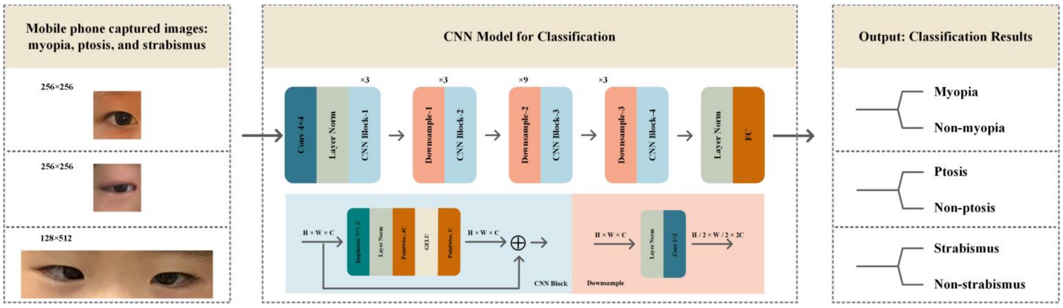

Abbreviation: AI, artificial intelligence; CNN, convolutional neural networks.

© 2024 Shu Q et al. *JAMA Network Open.*

eFigure 2. Performance of myopia, strabismus, and ptosis detection models. Receiver operating characteristic (ROC) curves and area under the curve (AUC) are shown for (A) myopia, (B) strabismus, and (C) ptosis.

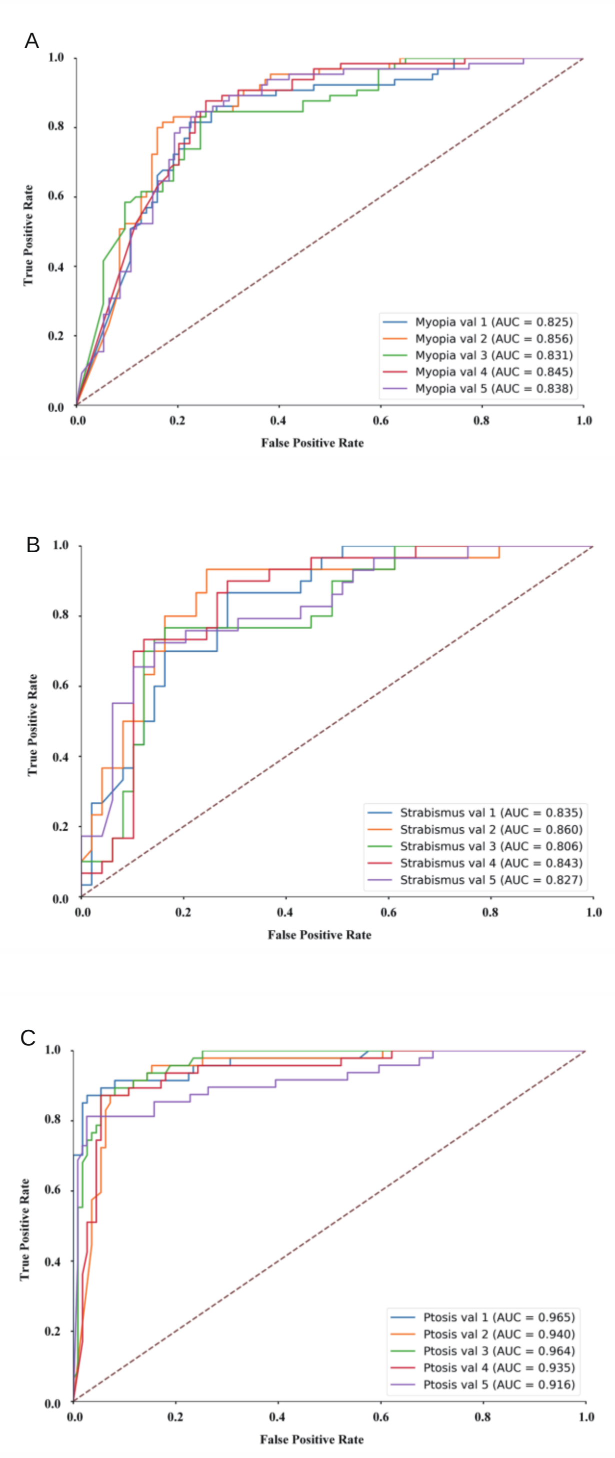

© 2024 Shu Q et al. *JAMA Network Open.*

eFigure 3. Comparison of the classification performance of the model on the testing dataset.

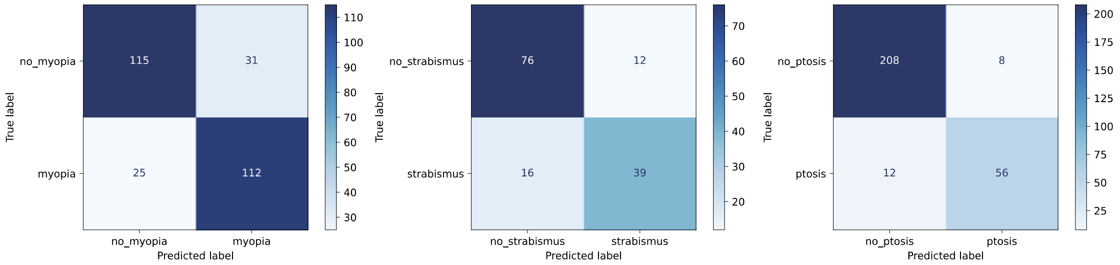

Legend: The results for myopia, strabismus, and ptosis are shown as a confusion matrix in which the vertical axis represents the true labels and the horizontal axis represents the predicted labels.

© 2024 Shu Q et al. *JAMA Network Open.*

eFigure 4. Pediatric eye diseases detection APP design.

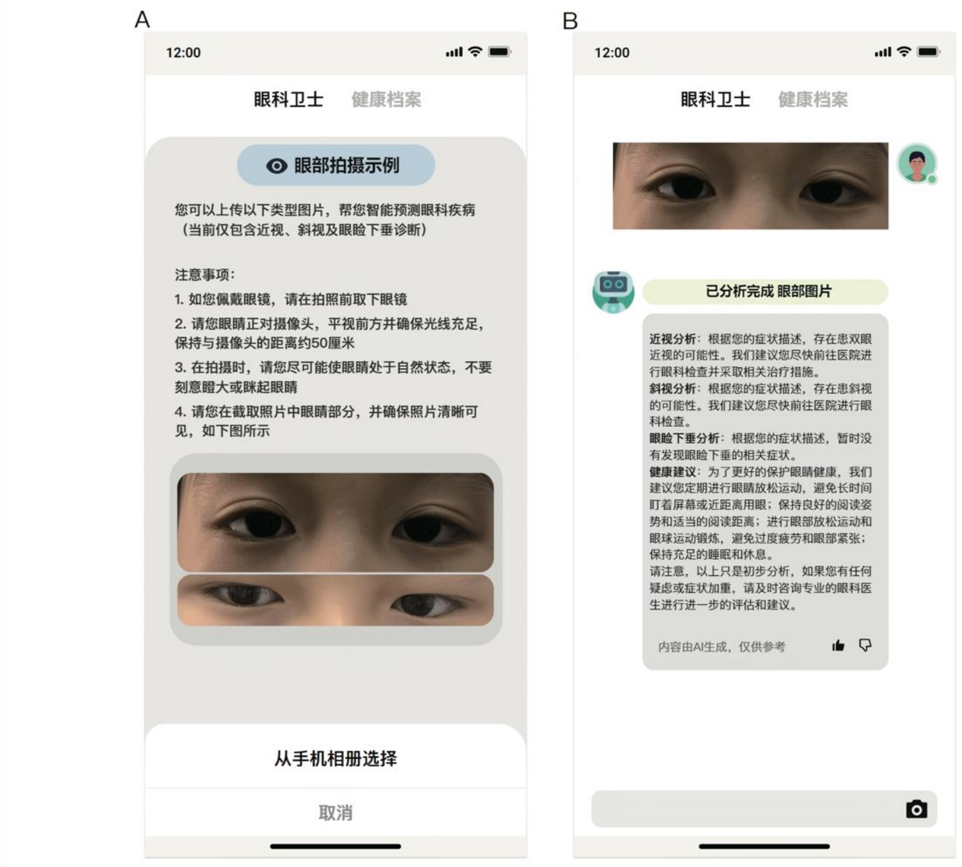

Legend: A. Notifications before taking a picture for prediction. Translation: 1. If you are wearing eyeglasses, remove them before taking the picture. 2. Place your eyes directly in front of the camera, keeping the distance from the camera at about 50 centimeters/1.64 inch. 3. Look straight ahead and make sure there is enough light. 4. Keep your eyes as natural as possible when taking the picture, do not widen or squint your eyes. 5. Use a screenshot tool to capture the eye part and make sure the photo is clear. **B.** Prediction of the uploaded picture. Translation: Myopia analysis: According to your symptom, there is a possibility of binocular myopia. We recommend that you go to the hospital as soon as possible for an ophthalmologic examination and take relevant treatment measures. Strabismus analysis: According to your symptom description, there is a possibility of strabismus. We recommend that you go to the hospital as soon as possible for an ophthalmologic examination. Ptosis analysis: According to your symptom description, there are no symptoms of ptosis for the time being. Health suggestion: In order to better protect your eye health, we recommend that you perform regular eye relaxation exercises to avoid staring at the screen for a long period of time or using your eyes in close proximity. Besides, it is necessary to maintain a good reading posture and appropriate reading distance; and maintain sufficient sleep and rest.

Please note that the above is only a preliminary analysis. If you have any concerns or symptoms worsening, please consult a professional ophthalmologist for further assessment and advice.

© 2024 Shu Q et al. *JAMA Network Open.*
