# Supplementary material for: Artificial Intelligence for Early Detection of Pediatric Eye Diseases Using Mobile Photos
Source: JAMA Netw Open. 2024 Aug 6;7(8):e2425124. doi: 10.1001/jamanetworkopen.2024.25124 (PMC11304122; doi:10.1001/jamanetworkopen.2024.25124)
Supplement: Supplement 2. — Data Sharing Statement [file jamanetwopen-e2425124-s002.pdf]

Data Sharing Statement

Shu. Artificial Intelligence for Early Detection of Pediatric Eye Diseases Using Mobile Photos.
JAMA Netw Open. Published August 06, 2024. doi:10.1001/jamanetworkopen.2024.25124

Data
Data available: No
